# Improving dental care access for head and neck cancer patients in primary care: developing the Cancer Action Support Practice pathway in South West England

**DOI:** 10.1038/s41415-026-9634-6

**Published:** 2026-05-22

**Authors:** Alexander J. Pollard, Claire Forbes-Haley, Joanne Purvis, Terrance Chikurunhe, Matthew Jerreat

**Affiliations:** 851554969383447784555 Torbay and South Devon NHS Foundation Trust, Torbay, UK; South West Restorative Managed Clinical Network, NHS England, UK; Bristol University Dental School, Clinical Trials Unit, Bristol, United Kingdom; 806299203306683243929 University Hospitals Plymouth, Plymouth, UK; NHS England, England, United Kingdom; 896035453014369073833 South West Restorative and Paediatric Dentistry Managed Clinical Networks, NHS England, United Kingdom; 679379445774538351294 South West Collaborative Hub (Hosted by Somerset ICB), NHS England, United Kingdom; 753409918917508507663 NHS England, England, United Kingdom

## Abstract

**Aims** Describes the development and early implementation of the Cancer Action Support Practice (CASP) pathway in South West England, aiming to improve dental care access for head and neck cancer (HNC) survivors in the primary care setting.

**Summary of CASP development** Stakeholder engagement was central to CASP's design, involving input from general dental practitioners, local dental committees, commissioners, and secondary care teams. Funding and commissioning options were explored through the regional integrated care boards (ICBs). CASP aligns with national policy, including NHS England's guidance on oral health for cancer patients. CASP provides a structured pathway enabling HNC patients to access routine and preventive dentistry in primary care to prepare them for and maintain them after oral rehabilitation. Two commissioning models (units of dental activity uplift and sessional rate approach) were proposed, allowing regional flexibility by commissioning ICBs. Consultant-led peer review, data collection, and ongoing professional development are embedded. A pilot CASP has been commissioned in Cornwall, with further strong regional interest.

**Conclusion** CASP demonstrates a regionally coordinated approach to improving dental access for a vulnerable and complex patient group. The model supports integration between primary and secondary care, informed by national guidance and local clinical need, and has potential applicability to other medical conditions with significant dental involvement.

**Supplementary Information:**

Zusatzmaterial online: Zu diesem Beitrag sind unter 10.1038/s41415-026-9634-6 für autorisierte Leser zusätzliche Dateien abrufbar.

## Key points

- Head and neck cancer patients are a high risk group, especially in the context of radiotherapy treatment involving the jaws.
- Access to a dental home is extremely important for these patients, to avoid deleterious outcomes while using NHS resources appropriately.
- Cancer action support practices are an example of a regionally coordinated approach to improve dental access for this vulnerable group.

**Supplementary Information:**

Zusatzmaterial online: Zu diesem Beitrag sind unter 10.1038/s41415-026-9634-6 für autorisierte Leser zusätzliche Dateien abrufbar.

## Introduction

Head and neck cancer (HNC) represents a significant healthcare burden, with approximately 12,200 new diagnoses annually in the UK.^1^ Despite reductions in traditional risk factors like smoking, over the past decade the incidence of HNC has continued to rise, which is largely being driven by human papillomavirus-related cancers that effect a younger population.^1^^,^^2^ Treatment typically involves complex combinations of surgery, radiotherapy, and chemotherapy, often resulting in severe functional and/or aesthetic impairments including trismus, xerostomia, and osteoradionecrosis.^2^^,^^3^^,^^4^

In the South West region specifically, there are estimated to be nearly 10,000 HNC survivors, with up to 70% having undergone radiotherapy.^5^ Dental access in this region remains below the national average, exacerbating the already complex dental needs of these patients. Adult NHS dental access in the South West was reported as low as 47.3% between 2019–2020, significantly impacting vulnerable patient groups such as those recovering from HNC.^6^

In an effort to address some of these challenges, the South West Restorative Managed Clinical Network (MCN) considered adaptation of a model developed by the national paediatric dental MCNs for child-friendly dental practices. Consideration was given to adopting a similar commissioning system to develop a pathway to support cancer patients. The South West Oncology Restorative Network (SWORN) was established in 2023 and became part of the South West MCN for restorative dentistry. SWORN serves to enhance collaboration, share expertise, and improve clinical outcomes through education and regional audits/quality improvement projects/research. SWORN has helped to create a united voice for South West restorative services involving head and neck cancer patients and has become a key stakeholder group in the development of the Cancer Action Support Practice (CASP) pathway. Through focus groups at their annual meeting, SWORN agreed that it was essential to improve access to primary dental care for head and neck cancer patients. This was also supported by national strategies such as the chief dental officer's guidance on oral healthcare provision for cancer pathways.^7^ This paper underpins regional initiatives and highlight the critical role primary care dentistry can play in supporting these patients.

## Development of cancer action support practices

The CASP pathway was developed through a coordinated effort involving members of the South West restorative MCN and the regional chief dental office. This structured pathway provides essential routine dentistry, preventive care, and cancer surveillance for HNC patients post-treatment, effectively providing a parachute between secondary and primary care for patients that don't have a primary care dental home. The focus of CASP was to allow secondary care restorative units to work seamlessly with primary care dental support for their patients. The support could be accessed once an individual's complex hospital-based oral rehabilitation was completed, or where required to stabilise basic dental disease before oral rehabilitation. However, CASP does not play a role in prehabilitation of cancer patients, which should always be led by a consultant in restorative dentistry. The CASP pathway supports an open line of communication between CASP practices and secondary care restorative dentistry providers. Extensive stakeholder engagement was crucial. Lessons were learned through meeting with primary care dental teams and LDC chairs who advised on adjustments to the pathway by focusing on the realities of delivering CASP. Regular meetings took place with commissioners from integrated care boards (ICBs) and the collaborative commissioning hub, clarifying funding mechanisms and commissioning complexities. Conversations took place regarding financial risk and sustainability, which informed decisions regarding appropriate funding models. Stakeholders were regularly engaged through MCN meetings, both during virtual MCN meetings and by requesting written feedback on documents such as the service specification at other times. In addition, a smaller stakeholder working group met regularly to keep the project progressing. Meetings were chaired by one of the project leads with minutes recorded by the MCN network manager along with action points that were allocated to named individuals. Data collection and analysis was needed to provide realistic numbers of people with HNC who might need to access CASP. This information was not always routinely available, requiring audits and fact finding conversations with care providers to be undertaken.^5^ Some of these data were also available from regional dashboards on cancer.

Input from general dental practitioners (GDPs) significantly influenced CASP's practical implementation. GDPs shared critical insights about managing HNC patients in primary care, due to their complexity. This highlighted the administrative workload associated with a scheme like CASP, e.g., audit and triage, primary care support. Concerns were raised about the appropriateness of patient referrals to primary care if guidelines were unclear, reinforcing the necessity for transparent referral criteria to CASPs and scope for ongoing communication where additional guidance is required. Contributions from SWORN were important for pathway construction. Workshop sessions at the SWORN annual meeting revealed potential gaps in existing care pathways, highlighted difficulties with communication between primary and secondary care, and demonstrated training requirements. SWORN also supported the plans for structured consultant led peer-review sessions, which are part of the CASP funding model. This peer review will help to strengthen regional training opportunities, enhancing clinician confidence and standardise care between CASPs. Discussions with members of Restorative Dentistry UK, particularly the Head and Neck Cancer Clinical Excellence Network, ensured CASP was grounded in robust, evidence-based clinical frameworks, aligning regional efforts with national best practices.^2^

## Commissioning and contracting

Central to CASP's implementation was use of an appropriate funding model. Two main commissioning models emerged:

- Units of dental activity (UDA) uplift model:
  - Pros – flexible scheduling aligned with treatment complexity
  - Cons – potential financial unpredictability due to variable treatment times and envisaged high treatment need
- Sessional rate model:
  - Pros – predictable scheduling and guaranteed availability of dental appointments
  - Cons – risk of under-used appointment slots, creating inefficiencies. CASP practices unable to undertake lab work as this would not be covered by the payment structure.

Following detailed analysis and stakeholder consultations, the commissioning approach prioritised a hybrid model, allowing the individual ICBs to select between a UDA uplift or sessional rate model. Following the process set out in the South West an initial concept paper was presented to a governance group that reviews and feeds back on all proposed new dental initiatives under consideration. Feedback from the ICBs suggested that they wanted to understand data capture of CASPs for quality assurance and optimisation of resource usage, as well as detailed explanations around peer review. Final amendments were made having regard to each South West ICB around patient numbers, proposed data capture from CASPs, and provision of a clear financial structure for peer review. The paper was then re-submitted for presentation to the South West Primary Care Oversight Group for a decision and final approval. Supporting documents (see online Supplementary Information) were developed following final review and expressions of interest to be launched regionally for use with CASPs:

- Referral form
- Referral guidance
- Patient information leaflet
- Data collection form.

Interest was shown from Cornwall ICB which have successfully recruited a pilot CASP using the sessional rate model, initially allowing the practice to use some of their already commissioned sessional time to manage CASP patients. The pilot CASP is collecting data that will then allow refinement of the commissioning model by providing greater understanding of patient numbers and complexity, as well as treatment time and cost factors. Other areas are currently on hold due to National commitment to urgent care priorities, and NHS re-organisation. However several ICBs have signalled their intent to commission CASPs in their areas. Peer-review provision was embedded within the CASP commissioning model, involving restorative dentistry consultants offering continuous support, training, and quality assurance. This peer-review element was integral to the costing strategy, ensuring clinical standards while providing ongoing professional development for primary care dental teams. SWORN is another pathway for regional practitioners to access HNC-related continued professional development and a yearly face-to-face meetings.

## Implementation, anticipated impact, and follow-up

**Fig. 1:**
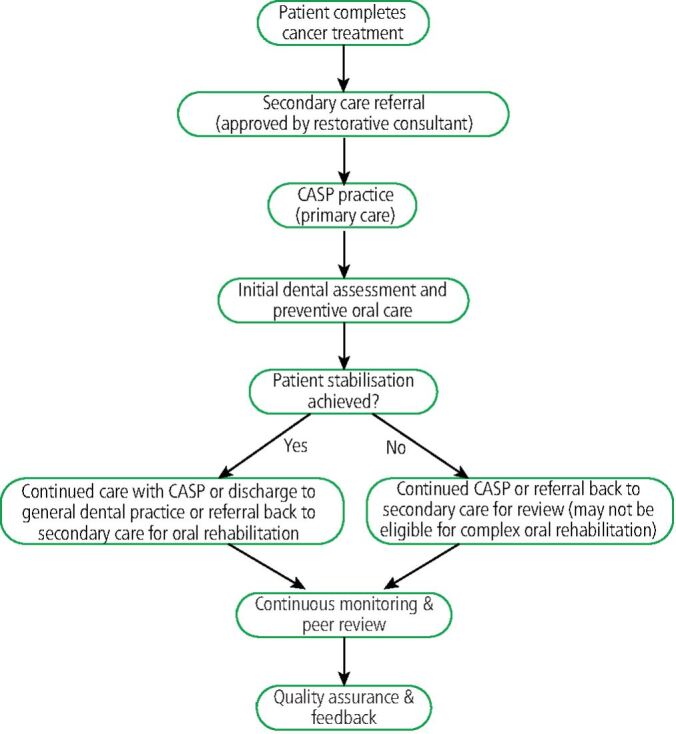
Cancer Action Support Practice patient referral and treatment pathway. *Stability refers to reduction of related risk factors and stabilisation of active carious lesions and periodontal diseases

CASP is expected to significantly enhance primary dental care access for HNC patients. It is hoped that it will reduce long-term complications, remove primary care from secondary care, improve patient experience and standardise care quality across the region. Initial pilot implementation for two years will carefully monitor clinical activity, patient experience, outcomes/satisfaction, and economic sustainability. Regular review meetings and audits will be required to ensure accountability, transparency, and continuous improvement. Each practice involved in CASP will be required to complete a FP17 form along with a Microsoft data collection form on a quarterly basis. This will help to evaluate the overall effectiveness of the programme. Engagement in peer review monthly meetings and audit will be required to address clinical issues and provide support to those involved. The current pathway from secondary care to CASP is via an electronic referral form. From the end of 2026 this will be replaced with digital electronic referrals directly from secondary care to primary care in line with the ten-year plan (see Fig. 1).

## Conclusion

The development of CASP demonstrates the potential for integrated regional initiatives to meaningfully improve care for complex patient groups with complex medical and dental care requirements. By strategically leveraging existing networks, aligning with national guidelines, and engaging stakeholders at all levels, this pathway provides a replicable model to enhance oral healthcare access for HNC patients nationwide. This model also has the potential to be used to support other medical specialities where dental care is paramount to achieving patient health.

## Supplementary Information

Supplementary Information 1-4 (PDF 304KB)
