# Supplementary material for: Improving dental care access for head and neck cancer patients in primary care: developing the Cancer Action Support Practice pathway in South West England
Source: Br Dent J. 2026 May 22;240(10):695–7. doi: 10.1038/s41415-026-9634-6 (PMC13197220; doi:10.1038/s41415-026-9634-6)
Supplement: Supplementary file 1 — Supplementary Information 1-4 (PDF 304KB) [file 41415_2026_9634_MOESM1_ESM.pdf]

Appendix 1 - Referral Form

Referral Form

Cancer Action Support Practice Pilot Referral Form

ALL sections MUST BE FULLY completed

A copy of patient's medical history must be included along with any additional supporting information

Referring Practitioner

Name and role of person completing referral:
Department Address:<br><br>Postcode:
Telephone Number:
Email Address:

Patient Information

Title:	Address:
Forename(s):	
Surname:	
Date of Birth:	Postcode:
NHS Number:<br>(If known)	Gender:
Telephone Contact Home	Mobile:
Additional Info:	
**General Medical Practitioner Information**:<br>Name:<br>Address<br><br>Postcode:	
Additional Medical / Social Information:	

**Reason for Referral (Please tick)**<br>**Routine** : Yes ☐ No ☐<br>HNC patients who requires a dental home following their oral rehabilitation	**Prompt**: Yes ☐ No ☐<br>HNC patients whose oral rehabilitation is to be delayed until they are dentally fit	**Urgent** : Yes ☐ No ☐<br>Patients with HNC who require basic dentistry to be performed prior to radiotherapy<br>or oncological surgery

What dental treatment / prevention strategies are required?<br>Oral hygiene instruction:	Yes ☐ No ☐
Dietary analysis / advice:	Yes ☐ No ☐
Fluoride prescription (5000ppm/1.1% NaF):	Yes ☐ No ☐
Dry mouth support required (if yes see clinic letter for details)	Yes ☐ No ☐
Support due to limited mouth opening/access	Yes ☐ No ☐
TMJ range of movement exercises	Yes ☐ No ☐
Teeth effected by radiotherapy (if yes see clinic letter for details)	Yes ☐ No ☐
Supragingival PMPR:<br>Non-surgical management of periodontitis (steps 1-4 as per BSP guidance documents)	Yes ☐ No ☐<br>Yes ☐ No ☐
Direct restorations	Yes ☐ No ☐
Root canal filling	Yes ☐ No ☐
Other – please specify:	

Radiographs included:<br>Date taken:	Yes ☐ No ☐
Request for radiographs to be taken:<br>Please specify:	Yes ☐ No ☐
Does the referring department need to review this patient again?<br>Refer back when dentally fit<br>Review appointment arranged.	Yes ☐ No ☐<br>Yes ☐ No ☐<br>Date:
Patient discharged from Secondary Care unless further review requested	Yes ☐ No ☐
**Declaration**<br>Referring Practitioner - I have explained to the patient that he / she is now being referred to Cancer Action Support Practice<br>Cancer Action Support Practice Information Leaflet/QR Code given	Yes ☐ No ☐<br>Yes ☐ No ☐
Signed:<br>Designation:<br>Date:<br>(sent back to regional RD Consultant via nhs.net email if required Yes ☐ No ☐ N/A ☐	
Signed and agreed by Restorative Consultant, name:<br>Print name:<br>Date:<br>(sent back to regional RD Consultant via nhs.net email if required Yes ☐ No ☐ N/A ☐	
**Sent to CASP practice via nhs.net email** Yes ☐ No ☐	

**Date Sent:**

Outcome following referral -To be completed by CASP

Patient accepted	Yes ☐ No ☐<br>If no, reason why?
Patients Home Postcode:	
CASP Postcode:	
Referring Secondary Care Postcode:	
Additional comments:	
Triaged by:	
Date Email Received:	
Name of CASP:	

Appendix 2 - Referral Guidance

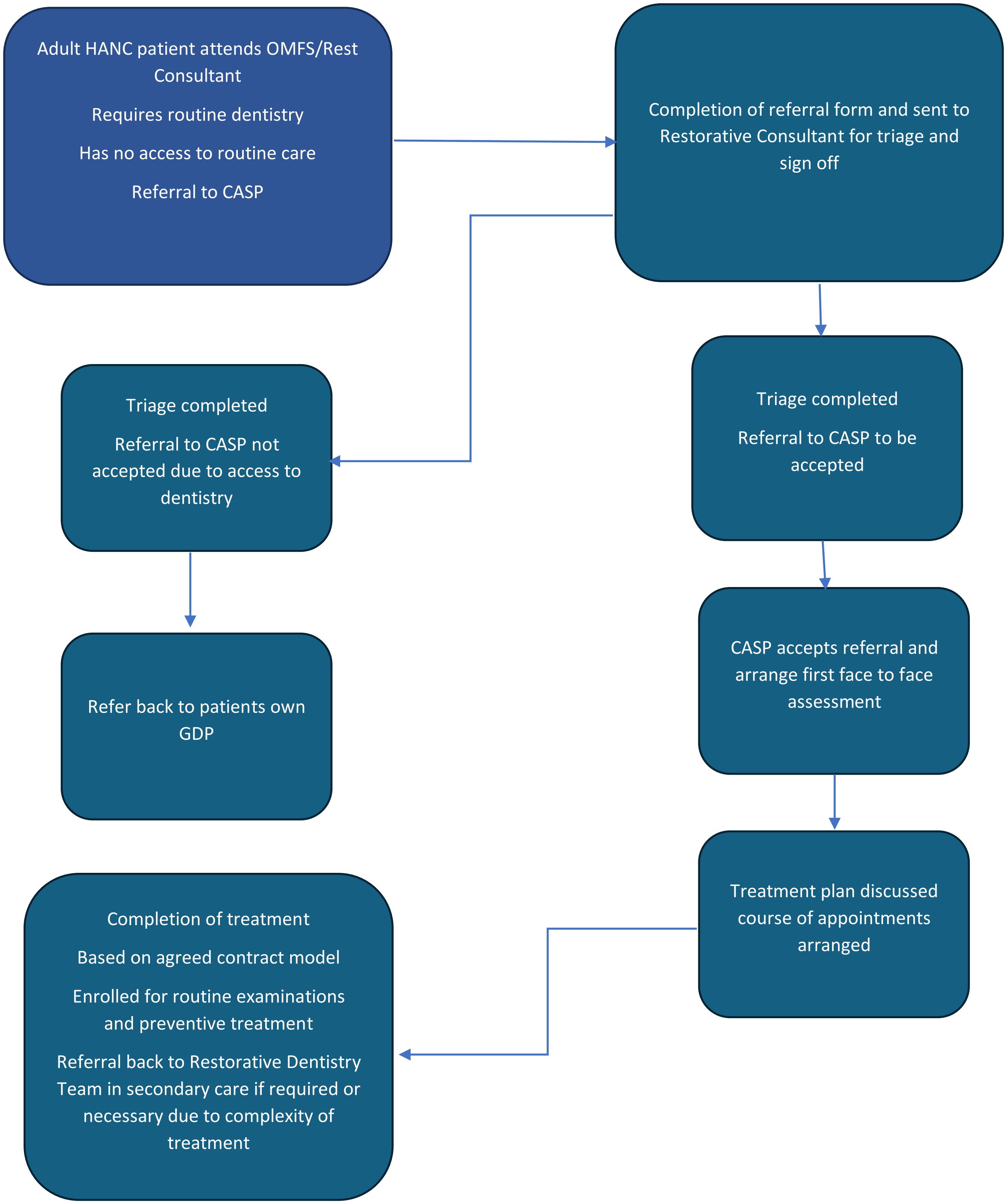

Appendix 3 - Patient Information Leaflet

Complaints

Our aim is to have satisfied patients who are pleased with their experience under our care. If you are not entirely satisfied with any aspect of your care, bring this to the attention of the treating clinician or nurse and every endeavour will be made to resolve your concerns as quickly as possible.

How to look after your teeth

Remember…

- Brush your teeth twice a day last thing at night, and one other time of the day.
- Use a small headed toothbrush and a fluoride toothpaste. If you have been advised to use a high strength toothpaste, please ensure you have access to a prescription from your dentist or doctor.
- Your toothbrushing routine should take 2—3 minutes.
- For best results always spit, **do not** rinse with water.
- Cleaning between the teeth with interdental brushes or floss at least once a day.
- Try having sugary drinks and foods less often, keep them to meal times.
- Eat a balanced, healthy diet with at least five portions of fruit and vegetables a day.
- Stop smoking and cut down on the amount of alcohol you drink.

V1.3/Dec24/CASP

NHS England South West

Cancer Action Support Practice Service Leaflet

South West Restorative MCN

Introduction

You have been given this leaflet to help provide answers to some of the questions you may have regarding your referral to a Cancer Action Support Practice. If you have any further questions, write them down and you will be able to discuss them with the dental clinician during your appointment.

What is a Cancer Action Support Practice?

A Cancer Action Support Practice (CASP) is an NHS Primary Care Dentist who supports those patients who have completed treatment for Head and Neck Cancer Treatment from a hospital setting and do not have access to a dentist.

NHS dentistry refers to the care of patients requiring the restoration and maintenance of their oral and dental tissues encompassing the following specialties:

- Operative dentistry: the filling of teeth.
- Periodontology: involves the prevention and treatment of gum disease.
- Endodontics: includes root canal treatment.
- Prosthodontics: involves the replacement of missing teeth.

Why am I being referred to CASP?

You have been referred to CASP as we understand you do not currently have access to a dentist. The CASP will provide you with routine dental treatment along with general oral health prevention.

When will I receive an appointment?

Your referral will be completed and shared with a CASP closest to your home postcode. The referral will be triaged by the practice and a member of staff will contact you directly via the telephone number you have provided. As part of your referral a treatment plan will be provided by the hospital.

An initial assessment appointment will be offered which will be give you the opportunity to ask any questions and discuss the course of treatment required.

What can I expect at the first appointment?

On your first visit, it will be for initial assessment, diagnosis and confirmation of a treatment plan. You will be assessed by a dental clinician who will review your dental history and any future plans. Following an initial discussion , a full examination will be undertaken. X-Rays may be taken if required and you will be asked to sign a written consent form. Results of the examination will be discussed and treatment options explained. Your appointment may take approximately 30 minutes.

Should I bring anything to my first appointment?

Any information relating to your medical conditions together with a list of medications. If you are taking Warfarin, bring your yellow record (INR) book along with any medical alert books. If you have any dentures or mouthguards bring these with you along with any x-rays or notes.

If I require treatment who will carry this out?

After your consultation with the dentist your treatment plan will be discussed and agreed. Your treatment may be completed by either of the following:

- Dentist
- Postgraduate Dentist
- Dental Therapist / Dental Hygienist
- Dental Nurse

What to expect during treatment?

There may be a wait following the first appointment which will be dependent on the availability of appointments and the urgency of the treatment required. Some treatment plans can be complex and may take a few visits. Emergency service is not available. However, as a patient of the practice if you are in pain please contact them directly.

What happens when treatment is complete?

On completion of your treatment, you will be advised whether a regular follow-up with the dentist is required. You will remain with the CASP for a number of visits, however, this will not be your long term dental home and you will advised to continue to look for a permanent dental practice following final discharge.

Appendix 4 - Data Collection Form

Date of monthly submission	Name of contract holder	Contract number	No: of programme treatment sessions / appts carried out per month	No: of patients attending for 1st appt per month	No: of patient's ref per month by HANC service	No: of trmt appts (incl hyg) per month	No: FTA late cancellation less than 24hrs	Admin time in hours (outside of clinical time)	No: patients referred to smoking cessation services	No: patients referred to alcohol/drug use services	Comments or additional info
